# Correlations between IL-6 serum level and olfactory dysfunction severity in COVID-19 patients: a preliminary study

**DOI:** 10.1007/s00405-021-06868-5

**Published:** 2021-05-13

**Authors:** Luigi Angelo Vaira, Andrea De Vito, Giovanna Deiana, Chiara Pes, Federica Giovanditto, Vito Fiore, Jerome R. Lechien, Serge-Daniel Le Bon, Sven Saussez, Giordano Madeddu, Sergio Babudieri, Antonio Pazzola, Franco Bandiera, Alessandro Giuseppe Fois, Andrea Fausto Piana, Claire Hopkins, Giacomo De Riu

**Affiliations:** 1 Maxillofacial Surgery Operative Unit, Department of Medical, Surgical and Experimental Sciences, University of Sassari, Viale San Pietro 43/B, 07100 Sassari, Italy; 2 Biomedical Science PhD School, Biomedical Science Department, University of Sassari, Sassari, Italy; 3 Infectious and Tropical Diseases Unit, Department of Medical, Surgical and Experimental Sciences, University of Sassari, Sassari, Italy; 4 Clinical Epidemiology and Medical Statistics Unit, Department of Medical, Surgical and Experimental Sciences, University of Sassari, Sassari, Italy; 5 Internal Medicine Department, University Hospital of Sassari, Sassari, Italy; 6 Neuro-COVID Department, University Hospital of Sassari, Sassari, Italy; 7 Onco-COVID Department, University Hospital of Sassari, Sassari, Italy; 8 COVID-19 Task Force of the Young-Otolaryngologists of the International Federation of Oto-Rhino-Laryngological Societies (YO-IFOS), Paris, France; 9 Department of Human and Experimental Oncology, Faculty of Medicine UMONS Research Institute for Health Sciences and Technology, University of Mons (UMons), Mons, Belgium; 10 Department of Otorhinolaryngology, CHU Saint-Pierre, Brussels, Belgium; 11 Respiratory Diseases Operative Unit, University Hospital of Sassari, Sassari, Italy; 12 King’s College, London, UK

**Keywords:** COVID-19, IL-6, Interleukin 6, Smell, Anosmia, Cytokine, SARS-CoV-2, Coronavirus, Cytokine storm

## Abstract

**Background:**

Interleukin 6 (IL-6) is a proinflammatory cytokine that is secreted by cells infected with severe acute respiratory syndrome coronavirus 2 (SARS-CoV-2) and it is widely recognized as a negative prognostic factor. The purpose of this study was to analyze the correlations between the olfactory scores determined by psychophysical tests and the serum levels of IL-6 in patients affected by coronavirus disease 2019 (COVID-19)

**Methods:**

Patients underwent psychophysical olfactory assessment with Connecticut Chemosensory Clinical Research Center test and IL-6 plasma level determination within 10 days of the clinical onset of COVID-19.

**Results:**

Seventy-four COVID-19 patients were included in this study. COVID-19 staged as mild in 34 patients, moderate in 26 and severe in 14 cases. There were no significant differences in olfactory scores across the different COVID-19 severity groups. In the patient series, the median plasma level of IL-6 was 7.7 pg/mL (IQR 3.7–18.8). The concentration of IL-6 was found to be significantly correlated with the severity of COVID-19 with a directly proportional relationship. The correlation between IL-6 plasma concentrations and olfactory scores was weak (*r*_s_ = 0.182) and not significant (*p* = 0.12).

**Conclusions:**

In COVID-19 patients, psychophysical olfactory scores did not show significant correlations with the plasma levels of a well-recognized negative prognostic factor such as IL-6. This observation casts some shadows on the positive prognostic value of olfactory dysfunctions.

## Introduction

Olfactory dysfunctions are one of the most frequent clinical manifestations of coronavirus disease 2019 (COVID-19), affecting more than 70% of patients infected with SARS-CoV-2 [1–5].

Recently, the prognostic value of olfactory disorders has been the subject of heated debate. Although some researchers have found no correlation between the prevalence of loss of smell and severity of COVID-19 [6–9], many report that olfactory dysfunction is more frequent in mild forms [10–13] and some postulate that this is the price to pay for a more effective immune response against the virus in the olfactory epithelium [14].

Interleukin 6 (IL-6) is a proinflammatory cytokine that is secreted by cells infected with SARS-CoV-2 [15]. IL-6 is one of the factors behind the cytokine storm that occurs in the most severe cases of COVID-19 and serum levels of this cytokine are therefore a widely recognized negative prognostic factor [16–18]. Before the pandemic, only a few authors have investigated the correlation between the severity of chronic olfactory disorders and serum levels of IL-6, noting a statistically significant directly proportional correlation [19, 20]. These results support a hypothesis that IL-6 may play a role in biochemical pathological process underlying these chronic olfactory dysfunctions [19]. However, this directly proportional correlation conflicts with what has been proposed in COVID-19 patients in whom the severity of olfactory dysfunction appears to be inversely proportional to the severity of COVID-19 [10–13] and therefore likely inversely proportional to serum IL-6 levels, assuming that serum IL-6 levels largely reflect the severity of COVID-19 pneumonia and other major organ involvement.

The purpose of this study was therefore to analyze the correlations between the olfactory scores determined by psychophysical tests and the serum levels of IL-6 in patients affected by COVID-19 and admitted to the University Hospital of Sassari. We hypothesize that severity of olfactory dysfunction will be inversely proportional to serum IL-6 levels, which are a marker of COVID-19 disease severity.

## Materials and methods

This cohort observational study was conducted in the COVID departments of the University Hospital of Sassari (Infectious and Tropical Disease, Pneumology, Onco-COVID and Neuro-COVID operative units) between January 10 and February 1, 2021.

To be enrolled in the study, patients had to meet the following inclusion criteria: adults over 18 years of age, rhino-pharyngeal swab positive for SARS-CoV-2 infection, COVID-19 symptoms present for less than 10 days, patient acceptance for participation in the study. On the contrary, the study exclusion criteria were: uncooperative patients, assisted ventilation, psychiatric or neurological disorders, previous surgery or radiotherapy in the oral and nasal cavities, pre-existing self-reported smell and taste alterations, history of head trauma, allergic rhinitis, chronic rhinosinusitis.

All patients provided informed consent for participation in the study. The study protocol was approved by University Hospital of Cagliari Ethical Committee (PG/2021/5471).

Some clinical and epidemiological information was collected for all patients: age, gender and COVID-19 symptoms. All patients were followed up clinically until the nasopharyngeal swab was negative. The overall clinical severity of COVID-19 was classified according to Tian et al*.* [21] in mild, moderate, severe and critical.

Psychophysical olfactory evaluation was performed with the Connecticut Chemosensory Clinical Research Center test (CCCRC). The CCCRC is a validated, widely used and easy to perform psychophysical test. The methodology, the scoring system and its application in COVID-19 patients have been extensively described in previous studies [22–25]. The CCCRC includes the assessment of the olfactory threshold using solutions with increasing concentration of N-butyl acid and an identification task for common odorants. The olfactory score thus obtained allows to clinically classify the olfactory function in five categories: normal (scores 90 and 100), mild (scores 70 and 80), moderate (scores 50 and 60) or severe hyposmia (scores 20, 30 and 40) and anosmia (scores 0 and 10).

Within 24 h after the olfactory test, plasma levels of IL-6 (reference value < 5.9 pg/mL) were determined on a peripheral blood sample taken from each patient by means of a fully automated Elecsys system on a cobas e801 platform (Roche Diagnostics, Basel, Switzerland) as previously described [18].

The statistical analysis was performed with SPSS 26.0 (IBM, Armonk, NY, USA). Categorical variables are reported in numerals and percentages of the total. Descriptive statistics for quantitative variables are given as the mean ± standard deviation (SD) or median (interquartile range–IQR). The Kruskal–Wallis Test was performed to evaluate the statistical significance of differences in olfactory scores between clinical severity groups. Post hoc analysis with Mann–Whitney *U* test was used to define the different relationships of the olfactory severity score and IL-6 blood levels with each of the COVID-19 severity subgroups. The correlation between olfactory scores and IL-6 levels was assessed with the Spearman rank correlation coefficient. The level of statistical significance was set at *p* < 0.05 with a 95% confidence interval.

## Results

Seventy-four COVID-19 patients who met the inclusion and exclusion criteria were included in this study. Table 1 summarizes the epidemiological and clinical characteristics of the patients (Table 1).

**Table 1:** General and clinical features of the study population

Gender	
Male	47 (63.5%) [95% CI 51.5–74.4%]
Female	27 (36.5%) [95% CI 25.6–48.5%]
Age (years) Mean ± SD	63.4 ± 13.4 [95% CI 60.9–66.5]
Days from COVID-19 symptoms onset Mean ± SD	7.2 ± 3.2 [95% CI 6.5–7.9]
Clinical stage No. of patients (%)	
Mild	34 (46%) [95% CI 34.3–57.9%]
Moderate	26 (35.1%) [95% CI 24.4–47.1%]
Severe	14 (18.9%) [95% CI 10.7–29.7%]
Critical	0 (0%) [97.5% CI 0–4.7%]
Olfactory function assessment No. of patients (%)	
Normal	19 (25.7%) [95% CI 16.2–37.2%]
Mild hyposmia	11 (14.9%) [95% CI 7.6–25%]
Moderate hyposmia	18 (24.3%) [95% CI 15.1–35.7%]
Severe hyposmia	12 (16.2%) [95% CI 8.7–26.6%]
Anosmia	14 (18.9%) [95% CI 10.7–29.7%]

*95% CI* 95% confidence interval, *SD* standard deviation

COVID-19 was staged as mild in 34 patients, moderate in 26 and severe in 14 cases. At the time of psychophysical evaluation, 55 patients (74% of the cohort) presented with olfactory dysfunction including anosmia (14 cases; 18.9%), severe hyposmia (12 cases; 16.2%), moderate hyposmia (18 cases; 24.3%) and mild hyposmia (11 cases; 14.9%), the remainder having normosmia. Overall, the median olfactory score was 60 (IQR 30–87.5). There were no significant differences in olfactory scores across the different COVID-19 severity groups (Tables 2, 3).

**Table 2:** Olfactory scores and IL-6 level according to clinical severity of COVID-19

COVID-19 severity [21]	Mild (*N* = 34)	Moderate (*N* = 26)	Severe (*N* = 14)
CCCRC score Median (IQR)	60 (30–70)	50 (32.5–85)	85 (50–90)
Kruskal–Wallis test	*p* = 0.202		
IL-6 blood level (pg/mL) Median (IQR)	4.29 (3.5–10.6)	10.5 (6–18.2)	31.5 (17.8–44.3)
Kruskal–Wallis test	*p* < 0.001		

*CCCRC* Connecticut Chemosensory Clinical Research Center test, *IL-6* interleukin 6, *IQR* interquartile range

**Table 3:** Post hoc analysis results

	Mann–Whitney *U* test
CCCRC score	
Mild versus moderate COVID-19	*p* = 0.787
Moderate versus severe COVID-19	*p* = 0.153
Mild versus severe COVID-19	*p* = 0.082
IL-6 blood level	
Mild versus moderate COVID-19	*p* = 0.008
Moderate versus severe COVID-19	*p* = 0.002
Mild versus severe COVID-19	*p* < 0.001

**Fig. 1:**
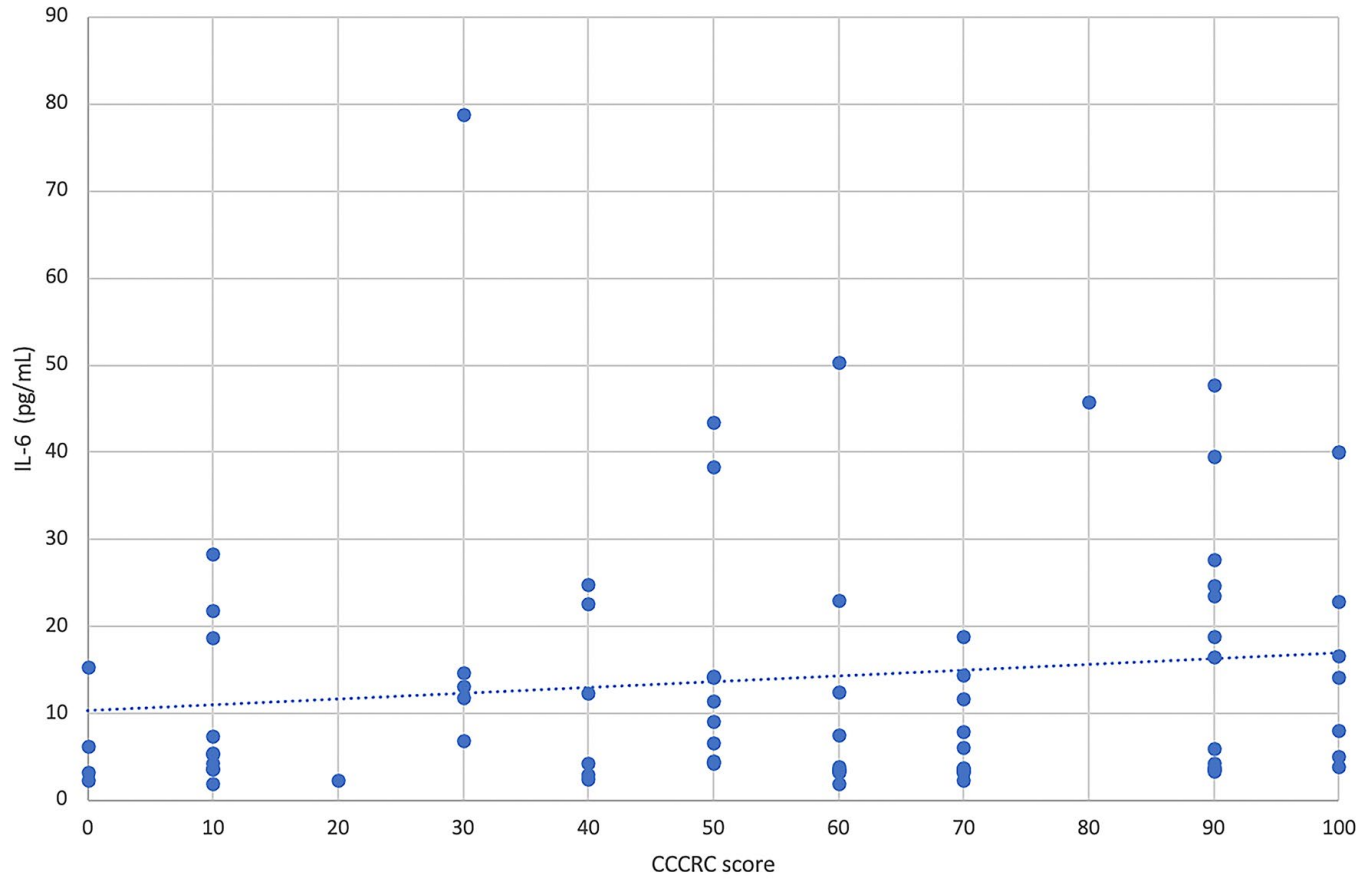
Correlation analysis between IL-6 plasma level and CCCRC scores

In the patient series, the median plasma level of IL-6 was 7.7 pg/mL (IQR 3.7–18.8). The concentration of IL-6 was found to be significantly correlated with the severity of COVID-19 with a directly proportional relationship (*p* < 0.001) (Table 2). Post hoc analysis with Mann–Whitney U test post hoc analysis showed that IL-6 blood concentration differences were significantly different across all COVID-19 severity subgroups analyzed (Table 3). The correlation between IL-6 plasma concentrations and olfactory scores was weak (*r*_s_ = 0.182) and not significant (*p* = 0.12) (Fig. 1).

## Discussion

To the best of our knowledge, only one article has previously evaluated the correlation between olfactory dysfunction in COVID-19 and IL-6 levels [26]. In this study, Cazzolla et al*.* reported a significant directly proportional correlation between IL-6 levels and the presence of self-reported olfactory dysfunction. This finding seems to be in line with what has been reported for other chronic inflammatory olfactory dysfunctions [19, 20] but, considering that high levels of IL-6 are a well-established negative prognostic factor [16–18], it would be in contrast to the fact that olfactory disorders seem to represent a favorable prognostic index in COVID-19 patients [10–13]. However, the study of Cazzolla et al*.* presents some major limitations which reduce the reliability of the results and that we have tried to overcome in the present study. First, the assessment of the olfactory function is not based on psychophysical tests and it has been shown that self-reported olfactory loss alone significantly underestimates the real prevalence of smell disorders in COVID-19 patients [27, 28]. Second, the temporal distance from the onset of symptoms was not considered among the inclusion and exclusion criteria. This is crucial if we want to establish whether the presence and severity of the olfactory disturbance at the onset of COVID-19 can correlate with other established prognostic indices. In this sense, a further limitation is that only mild and moderate COVID-19 patients were included in the study by Cazzolla et al*.* [26].

In our study, patients were evaluated within the first 10 days of symptom onset, when olfactory dysfunction should not yet have begun to recover [29, 30]. At this early stage of COVID-19, 74.3% of patients had olfactory dysfunction. This prevalence is in line with those reported by most authors [1, 3–5, 8, 13, 22, 23, 25]. The olfactory scores did not show significant differences between the various COVID-19 severity groups. Although most authors attribute a positive prognostic value to olfactory disorders [10–13], this finding has never been detected in patients belonging to the Italian centers who participated in our prospective cohort studies during the first wave [7, 23–25]. However, plasma levels of IL-6 were shown to be a reliable negative prognostic index, demonstrating a directly proportional and significant correlation with the severity of the clinical picture of COVID-19. The differences between the median IL-6 blood level were significant not only between patients with severe COVID-19 and those with mild and moderate forms but also between subjects with mild and moderate clinical pictures. This supports our hypothesis that serum IL-6 levels reflect the severity of major organ involvement. The correlation between olfactory scores and IL-6 levels was therefore weak and not significant.

Our findings give support to two main hypotheses. First, the prognostic value of olfactory disturbances appears to be significantly less strong than other clinical and laboratory data such as IL-6. The data presented in the literature to date are discordant, often retrospective and based on the only self-reported olfactory loss or with the need for hospitalization as the only prognostic outcome. Second, the pathogenesis of olfactory dysfunctions in COVID-19 is more likely caused by a local inflammatory [31–33] process than a systemic cytokine storm that then damages the olfactory epithelium. In this inflammatory process, it is not excluded that locally produced IL-6 may play a role in the genesis of olfactory dysfunction by acting on neurons [34] or glial cells [35] as occurs in other inflammatory and post-viral smell disorders [19, 36]. It would have been interesting to determine IL-6 levels in nasal mucus but this was not possible due to technical problems at the time of this study. However, any nasal production of IL-6 within the olfactory epithelium is likely insufficient to significantly contribute to serum IL-6 levels.

This study has some limitations. The number of patients included in the study is not yet sufficient to draw firm conclusions. While there is a risk we have rejected significant association between olfactory scores and IL-6 levels due to type 2 statistical error, we did have sufficient participants to detect a strong and statistically significant correlation between COVID-19 severity and IL-6 which is a well-recognized negative prognostic factor. In future studies, it would be useful to evaluate the correlations between olfactory scores and both serum and mucus levels of other pro-inflammatory cytokines such as IL-10, IL-12, IL-15 and TNF-α which are secreted by cells infected with SARS-CoV-2 [37] and involved in the cytokine storm at the base of the most severe cases of COVID-19 [38]. Moreover, it could be interesting to prospectively evaluate patients by monitoring the recovery of olfactory function and correlating it with IL-6 levels in the early stages of infection. It is well known that IL-6 can be both pro and anti-inflammatory and suppresses the production of TNF-α [39]. TNF-α is increased in the olfactory mucosa following SARS-CoV-2 infection and causes damage to olfactory sensory neurons (OSNs) [40]. Perhaps elevated systemic IL-6 leads to reduced TNF-α production in olfactory epithelium and limits damage to OSNs, therefore higher IL-6 early on could be associated with faster recovery of olfactory loss. We may not have been able to see this correlation as we tested patients in the earliest stages of the disease, during the phase of injury to the supporting cells but before the secondary loss of OSNs has occurred.

## Conclusions

In COVID-19 patients, psychophysical olfactory scores did not show significant correlations with the plasma levels of a well-recognized negative prognostic factor such as IL-6. This observation casts some shadows on the positive prognostic value of olfactory dysfunctions.
